# Co-formulation of IL-12 mRNA and doxorubicin in polymeric nanoparticles for simultaneous delivery in murine melanoma

**DOI:** 10.1039/d6pm00136j

**Published:** 2026-07-01

**Authors:** Elina Tanskanen, Hongning Sun, Kai-Chun Cheng, Jun Ishihara, Asha K. Patel

**Affiliations:** a National Heart and Lung Institute, Imperial College London London W12 0NN UK asha.patel@imperial.ac.uk; b Department of Bioengineering, Imperial College London London W12 0BZ UK

## Abstract

Liposomal or polymeric nanoparticles have been instrumental in improving the delivery of poorly soluble chemotherapeutics and those with dose limiting toxicity such as doxorubicin (DOX). More recently, nanoformulations have been shown to enable simultaneous delivery of emerging biomolecules such as siRNA. However, for larger nucleic acids such as mRNA, this remains challenging. In this study, we developed a poly(β-amino ester) (PBAE) based platform, capable of co-formulating mRNA and doxorubicin into nanoparticles. To demonstrate proof of concept using therapeutically relevant cargo, immunomodulatory interleukin-12 (IL-12) was selected as a model mRNA. IL-12 is a pro-inflammatory cytokine that promotes anti-tumour immunity partly through amplifying effector cytokines such as interferon-γ (IFNγ). We found that PBAE complexed DOX and mRNA into positively charged nanoparticles of 120 nm and size-exclusion chromatography indicated a DOX loading efficiency of over 97%. Co-association of both DOX and mRNA was characterised at a single nanoparticle level by nano-flow cytometry. Following delivery to B16F10 murine melanoma cells, more than 95% of cells were double-positive for DOX and Cy5-labelled mRNA, and confocal microscopy confirmed co-localised regions of DOX with mRNA. Interestingly, nanoformulated DOX had increased nuclear accumulation by 1.7-fold relative to free DOX, which correlated with a significantly reduced cell viability of 12.9% with PBAE-DOX/mRNA, compared to 26.6% for free DOX at the same dose. Moreover, despite this strong cytotoxic effect, reporter mRNA translation remained robust, with luciferase expression approximately two orders of magnitude above non-transfected controls at the highest DOX doses. Co-formulation of *IL-12* mRNA and DOX with PBAE demonstrated effective IL-12 protein secretion in transfected B16F10 cells with a simultaneous DOX dose dependent reduction in viability. Secreted IL-12 was bioactive, inducing dose-dependent STAT4 phosphorylation and IFNγ secretion in primary mouse splenocytes. Furthermore, in a syngeneic melanoma mouse model, intratumoural administration of PBAE-DOX/*IL-12* mRNA achieved significantly elevated levels of IL-12 and IFNγ in the tumour compared to the saline control, confirming delivery of DOX, as well as IL-12 protein secretion, and immunostimulatory activity *in vivo*. These findings demonstrate that PBAE is a promising platform for co-delivery of cytokine encoded mRNA with DOX in a single formulation, establishing feasibility for advanced chemoimmunotherapy approaches.

## Abstract 2

(1) PBAE nanoparticles complexed with DOX and IL-12 mRNA are endocytosed. (2) DOX localisation in the nucleus leads to apoptosis of cancer cells. (3) Concurrent translation of IL-12 mRNA is secreted from cytosol and stimulates T cells. Created with BioRender https://www.biorender.com/20en4jy.

## Graphical abstract

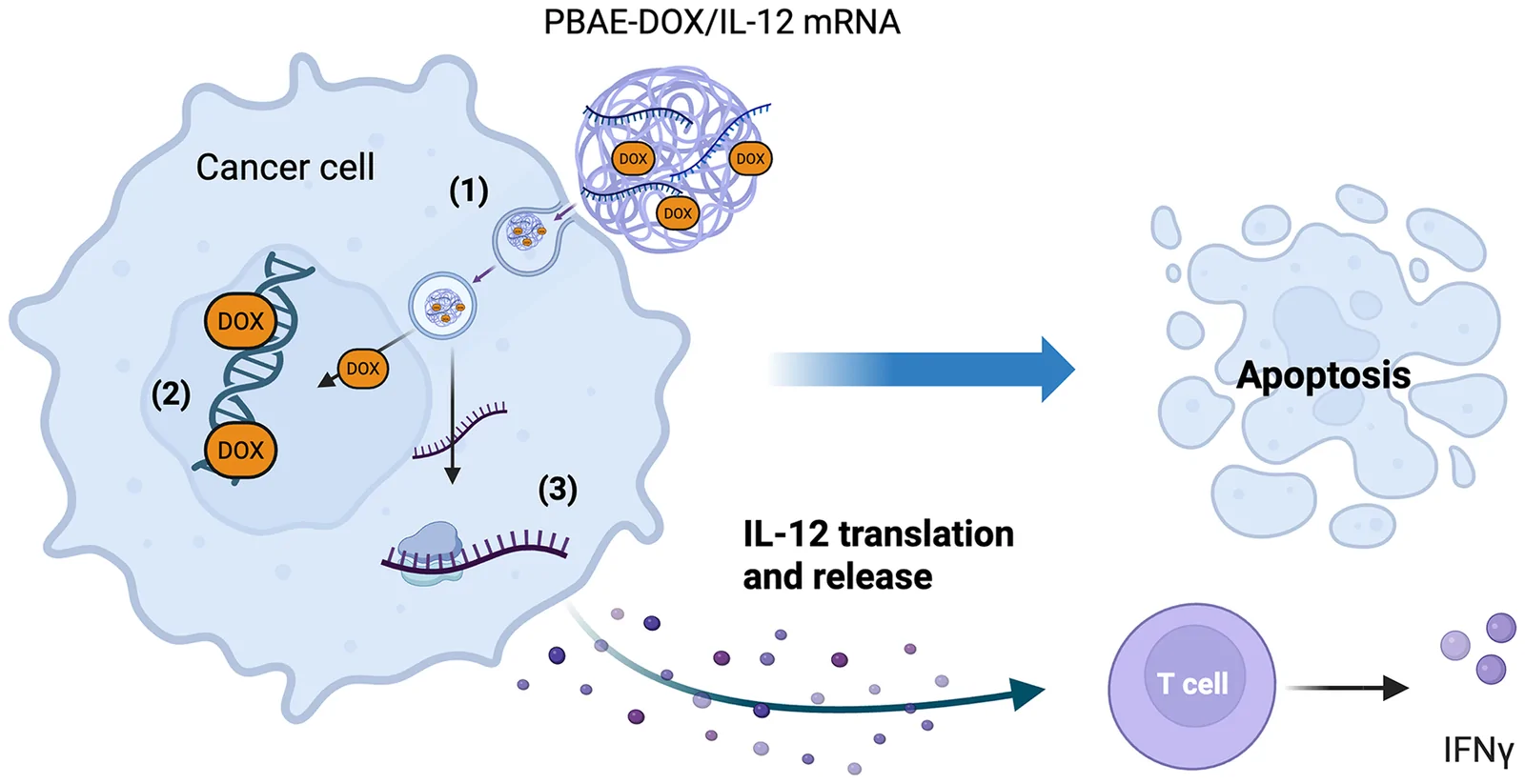

## 1. Introduction

Doxorubicin (Dox) is a cytotoxic anthracycline antibiotic, with chemotherapeutic applications across a range of solid and haematological malignancies.^1^ Nanoformulations, most notably PEGylated liposomal doxorubicin (Doxil), are commonly used to increase the therapeutic index,^2,3^ and recent advances in nanoparticulate systems have enabled co-formulation of doxorubicin together with other small molecule drugs, proteins, or nucleic acids that have been explored for synergistic effects.^4^ While co-delivery strategies for shorter nucleic acids such as siRNA or miRNA with doxorubicin have been reported,^5–10^ research into co-delivery of longer protein-encoding nucleic acids has focused on plasmid DNA.^11–14^ Messenger RNA is an attractive alternative to DNA, as it allows transient expression without the risk of insertional mutagenesis.^15^ However, mRNA is particularly difficult to deliver due to its high susceptibility to degradation, large size, and negative charge.^16^ Delivering mRNA within the same nanoparticle as DOX could allow for the transient expression of potentially any encoded therapeutic protein within tumour cells, while simultaneously inducing doxorubicin-mediated cell death.

Interleukin-12 (IL-12) is a pro-inflammatory cytokine that promotes Th1 transformation of the tumour microenvironment *via* stimulation of IFNγ signaling and recruitment of cytotoxic T cells. Delivery of IL-12 encoded as mRNA allows localised and sustained expression of IL-12 *in situ*, potentially overcoming issues such as systemic toxicity and short half-life of the cytokine.^17^ Consequently, there have been attempts to harness these benefits and intratumoural injection of *IL-12* mRNA has demonstrated potent anti-tumour effects in mouse models^18^ and safety in a clinical trial.^19^ Moreover, the co-delivery of *IL-12* mRNA with the small molecule drug docetaxel in lipid nanoparticles demonstrated the potential of combination strategies to enhance anti-tumour effects.^20^ Co-administration of doxorubicin and IL-12 protein has been shown to increase IFNγ accumulation and CD8+ T cell recruitment in the tumour,^21,22^ and increase M1 polarisation of macrophages,^23^ leading to enhanced anti-tumour effects. Therefore, exploring the co-delivery of *IL-12* mRNA and doxorubicin presents an attractive avenue for achieving synergistic effects; however, nanoformulation of *IL-12* mRNA with DOX has not yet been reported.

Poly(beta-amino esters) (PBAE) are a class of cationic, biodegradable polymers that can condense large anionic biomolecules such as DNA and mRNA into stable nanoparticles for local and systemic delivery in pre-clinical models.^24^ Additionally, PBAE can be synthesized from an array of chemically diverse monomers to control physicochemical properties such as hydrophobicity and charge density to enhance affinity for small molecules or allow direct conjugation for therapeutic application.^10^ PBAEs have been investigated for co-delivery of DOX with other therapeutic molecules for combination cancer therapy,^25–27^ but nanoformulation of mRNA and DOX has not yet been demonstrated.

The possibility of combining DOX with *IL-12* mRNA in a single nanocarrier is particularly compelling for immunologically cold tumours such as melanoma.^28^ Such co-delivery systems offer the potential to synchronise local exposure to two mechanistically distinct agents to enhance synergy. In this case, the translational machinery of melanoma cells is used to produce mRNA-encoded protein while simultaneously delivering a cytotoxic payload.

We hypothesized that polymeric nanoformulation of mRNA and DOX would enable simultaneous intracellular delivery for therapeutic protein production alongside chemotherapy induced cytotoxicity. In this study, we first sought to define formulation conditions that enable stable assembly of DOX and mRNA into PBAE nanoparticles. A successful co-delivery system must incorporate both cargos at sufficient doses that achieve physiological responses. To establish that cytotoxic DOX does not abolish mRNA function, we examined reporter mRNA translation under increasing DOX doses in a melanoma cell line and subsequently extended the system to therapeutically relevant *IL-12* mRNA. Finally, the platform demonstrated translational relevance *in vivo* in a tumour-bearing mouse model, establishing the feasibility of combining a conventional chemotherapeutic with protein-encoding mRNA within a single polymeric nanocarrier. These findings provide a foundation for future chemo-immunotherapeutic strategies.

## 2. Materials and methods

### 2.1 Materials

#### Biological reagents

EMT6 murine mammary carcinoma and B16F10 murine melanoma cell lines were purchased from ATCC. Dulbecco's phosphate buffered saline (D8537) and fetal bovine serum (F2442) were obtained from Sigma Aldrich. Dulbecco's modified Eagle's medium (DMEM) with GlutaMAX and 4.5 g L^−1^d-glucose (10566-016), RPMI1640 with GlutaMAX (61870-036) and 50 mM 2-mercaptoethanol (31350010) were obtained from Gibco. EZ Cap™ Firefly Luciferase mRNA (5-moUTP) (R1013), EZ Cap™ Cy5 Firefly Luciferase mRNA (5-moUTP) (R1010), and EZ Cap™ Mouse IL-12 mRNA (m1Ψ) (R1058) were obtained from Apexbio. Gel loading dye (B7024S) was purchased from New England Biosciences. RiboRuler High Range RNA Ladder (SM1821), T-PER™ Tissue Protein Extraction Reagent (78150) and Pierce™ BCA Protein Assay Kit (23225) were obtained from Thermo Scientific. A Bright-Glo™ Luciferase Assay System (E2610) and VivoGlo™ Luciferin (P1041) were obtained from Promega. Complete™ Protease Inhibitor Cocktail (04693116001) was purchased from Roche. PrestoBlue™ Cell Viability Reagent (A13261), ProLong™ Gold Antifade Mountant with DNA Stain DAPI (P36941), the mouse IL-12 p70 ELISA kit (88-7121-77) and the mouse IFN gamma ELISA kit (BMS606) were purchased from Invitrogen. The Mouse T Cell Activation/Expansion Kit (130-093-627) was obtained from Miltenyi Biotec. BD Phosflow™ Lyse/Fix Buffer 5× (558049), Phospho-STAT4 Alexa Fluor® 647 Mouse Anti-Stat4 (pY693), Anti-mouse CD3 BUV395 (740268), and Brilliant Violet 510™ anti-mouse CD8a antibody (100751) were purchased from BD Biosciences.

#### Chemical reagents

*N*,*N*-Dimethylformamide (anhydrous, 99.8%), bisphenol A glycerolate (1 glycerol/phenol) diacrylate (containing MEHQ as the inhibitor), 4-(2-aminoethyl)morpholine (99%), 1,5-diamino-2-methylpentane (99%), diethyl ether (99.0%, anhydrous, containing BHT as the inhibitor), triethylamine (99%), methanol (99.9%), dimethyl sulfoxide (anhydrous, 99.9%), 1 M magnesium sulfate solution (molecular biology grade) and Trizma® hydrochloride (99%) were purchased from Sigma-Aldrich. Doxorubicin hydrochloride (95.0%) and *N*-methyl-1,3-diaminopropane (98.0%) were purchased from Tokyo Chemical Industry UK Ltd. Calcium chloride (anhydrous granules) was purchased from Supelco Analytical Products. Pure sucrose was purchased from Scientific Laboratory Suppliers.

### 2.2 Preparation of PBAEs and hydrophobic doxorubicin

PBAE (branched DD90-118) was prepared as described previously.^29^ Bisphenol A glycerolate (1 glycerol/phenol) diacrylate, 4-(2-aminoethyl)morpholine, and *N*-methyl-1,3-diaminopropane were reacted at a ratio of 1 : 0.5 : 0.1 in anhydrous *N*,*N*-dimethylformamide (DMF) at a concentration of 150 mg mL^−1^ and stirred at 40 °C for 4 h, and then at 90 °C for 48 h. The solution was cooled to 30 °C and 1.5 molar equivalents of 1,5-diamino-2-methylpentane were added and reacted for 24 h at 30 °C. The solution was precipitated in cold anhydrous diethyl ether, and the product was collected by centrifugation at 3000*g* for 5 minutes and this washing step was repeated two more times. The precipitate was dried under vacuum overnight. Stock polymer solution was prepared in anhydrous dimethyl sulfoxide (DMSO) at a concentration of 100 mg mL^−1^ and stored at −80 °C.

Doxorubicin (DOX) was prepared from doxorubicin hydrochloride (HCl).^30^ Briefly, DOX-HCl was dissolved in methanol, and 2 molar equivalents of triethylamine (TEA) were added. The mixture was stirred at room temperature for 30 min. After that, the solvent and excess TEA were removed using a rotary evaporator. Doxorubicin was dissolved in anhydrous DMSO at a concentration of 10 mg mL^−1^ and stored at 4 °C.

### 2.3 Preparation of nanoparticles

PBAE and doxorubicin were dissolved in anhydrous DMSO, then added to mRNA diluted in PBS, or PBS only for controls, and mixed by pipetting to form nanoparticles. The PBAE : mRNA w/w ratio was kept constant at 50 : 1 for all experiments, and the concentration of doxorubicin was altered. For example, to prepare complexes at a 0.75 μg DOX dose, 2 mg mL^−1^ DOX in DMSO solution was added in equal volume to PBAE in 13.3 mg mL^−1^ DMSO. The DOX-PBAE solution was then diluted 1 : 20 v/v in a solution of mRNA in PBS at 7.02 ng μL^−1^, yielding 5 μg of PBAE, 0.75 μg of DOX and 100 ng of mRNA in a 15 μL volume.

### 2.4 Characterisation of nanoparticles by dynamic light scattering and NanoFCM

For DLS analysis, the particles were prepared as described in section 2.3. Briefly, DD90-118 stock solution and DOX were diluted in DMSO to obtain final concentrations of 3.3 mg mL^−1^ for PBAE and 0.67 mg mL^−1^ for DOX. FLuc mRNA (1 mg mL^−1^) was diluted with PBS to obtain 3.33 μg mL^−1^ mRNA solution. Subsequently, 5 μL of diluted PBAE-DOX solution was added to 100 μL of mRNA solution and incubated for 10 min. The nanoparticle suspension was transferred into a microcuvette for particle size measurement or diluted 1 : 10 in deionised water and transferred to a folded capillary cell for zeta potential measurement with a Malvern Zetasizer Pro.

Cy5-tagged FLuc mRNA was used to prepare the sample for NanoFCM analysis. As described above, the samples were diluted 10 times with PBS to obtain a dilution sufficient for detecting single-particle signals. A NanoFCM nanoanalyzer equipped with 488 nm and 640 nm lasers, and 488/10, 525/40, and 670/30 bandpass filters was calibrated using the manufacturer's quality control nanospheres. The histogram and mean fluorescence intensity of the 525 nm channel were used to characterise the loading of DOX, and the 670 nm channel was used to characterise the loading of Cy5-FLuc mRNA.

### 2.5 Quantification of doxorubicin-PBAE loading efficiency using size exclusion chromatography

The complexation of DOX with PBAE was characterised by size-exclusion chromatography (SEC) with Sephadex G-25 as the stationary phase in gravity-flow columns. The particles were prepared as described in section 2.4 and diluted 10 times before loading onto the column. Free DOX in DMSO (2 mg mL^−1^) was diluted in PBS as the control to measure the peak of non-complexed, free DOX. After equilibration with PBS, 100 μL of diluted sample was loaded onto a 3 mL column. Elution was performed using PBS, and the eluate fractions were collected every 0.2 mL. The concentration of doxorubicin was determined by measuring the absorbance at 495 nm. To quantitatively determine the encapsulation efficiency of DOX, the area under the curve (AUC) of peaks was calculated. The proportion of the AUC of the DOX loading peak to the total AUC was taken as the encapsulation efficiency.

### 2.6 Melanoma and breast cancer cell culture

B16F10 and EMT6 cells were cultured in 75 cm^2^ tissue culture flasks in DMEM GlutaMAX supplemented with 10% fetal bovine serum and 1% penicillin–streptavidin (penicillin: 100 units per mL, streptomycin: 100 µg mL^−1^). The cells were detached with 0.25% Trypsin-EDTA at 70–90% confluency for passage and plating for subsequent experiments.

### 2.7 Dose–response and cytotoxicity studies

1.2 × 10^4^ B16F10 and EMT6 cells were seeded on clear 96-well flat bottom plates and treated the next day with 100 ng of Firefly Luciferase mRNA and varying doses of free DOX or DOX-PBAE/mRNA at a 50 : 1 PBAE : mRNA (w/w) ratio. PBAE complexed with 100 ng mRNA (without DOX) and PBAE alone were delivered as controls. The media were replaced with fresh media after 4 hours. Cell proliferation was monitored for 72 hours using the CellCyte X live-cell imaging platform (Echo). Transfection efficiency was determined at 24 hours using the Bright-Glo™ Luciferase Assay System. Cell viability was measured at 0 h and 24 h using PrestoBlue™ Cell Viability Reagent and normalized against a non-treated control.

### 2.8 Quantifying uptake of doxorubicin and mRNA in cells using flow cytometry

5 × 10^4^ B16F10 cells were seeded in a 24 well plate. After 24 hours, the cells were treated with nanoparticles containing 100 ng Cy5-Firefly Luciferase mRNA and different doses of DOX (0.05–0.75 μg per well) for 4 hours or 0.75 μg per well for the free DOX group. Cells were collected and washed with PBS twice and the cellar uptake of doxorubicin and mRNA were tested using a BD Accuri C6 Plus.

### 2.9 Visualising doxorubicin and mRNA uptake and cellular localisation using confocal microscopy and imaging flow cytometry

8 × 10^4^ B16F10 cells were seeded on an 8-well Nunc™ Lab-Tek™ II Chamber Slide™ System (Thermo Fisher) and treated the following day with 200 ng of Cy5-tagged FLuc mRNA, and 400 ng of doxorubicin complexed with 10 μg of PBAE for 4 hours. At 6 h or 24 h, the cells were washed twice with DPBS and fixed with 4% PFA for 15 minutes. Following three more washing steps, the cells were mounted with ProLong™ Gold Antifade Mountant with DNA Stain DAPI. The cells were visualized using a Leica SP8 confocal microscope at 63× magnification. Nuclei were manually outlined using the DAPI channel with the DOX channel masked to prevent bias. DOX fluorescence intensity was quantified within each nuclear region of interest using Leica LAS X software.

For imaging flow cytometry, after 4 hours of treatment with nanoparticles, the cells were collected and washed twice with DPBS. Finally, the cells were resuspended in DPBS to a concentration of 1 × 10^7^ cells mL^−1^ and analysed using an ImageStream MK II imaging flow cytometer.

### 2.10 Translation and activity of IL-12 mRNA delivered within the PBAE-DOX/mRNA nanoparticles

1.2 × 10^4^ B16F10 and EMT6 cells were seeded on clear 96-well flat bottom plates and treated the next day for 4 hours with 100 ng of *IL-12* mRNA and varying doses of DOX encapsulated by PBAE at 50 : 1 PBAE : mRNA w/w. Cell culture media were assayed at 24 hours using mouse IL-12 p70 ELISA to quantify IL-12 translation. Primary mouse splenocytes harvested from C576B/L were cultured in RPMI 1640 media supplemented with 50 μM 2-ME and 10 ng mL^−1^ human IL-2 and activated using CD3ε and CD28-coated Anti-Biotin MACSiBead particles for 3 days. Activated splenocytes were rested without IL-2 for a day, seeded on a 96-well plate and treated with serial dilutions of cell culture media from PBAE-DOX/*IL-12* mRNA-transfected B16F10 cells. For the STAT4 phosphorylation assay, the cells were fixed in 1× BD Phosflow Lyse/Fix buffer after 15 minutes of stimulation and stained with Phospho-STAT4 Alexa Fluor® 647 Mouse Anti-Stat4 (pY693) and anti-mouse CD3 BUV395 before analysis with a Cytoflex flow cytometer. EC_50_ values were determined by nonlinear regression in GraphPad Prism 10 using a four-parameter logistic model ([agonist] *vs.* response – variable slope). For measuring IFNγ secretion, splenocytes were stimulated for 24 hours before cell culture media were assayed with mouse IFNγ ELISA.

### 2.11 *In vivo* imaging of PBAE-FLuc mRNA biodistribution

Female C57BL/6J mice were inoculated with 5 × 10^5^ B16F10 cells on the back skin. When tumours reached ∼50–200 mm^3^, they were intratumorally injected with PBAE-FLuc mRNA containing 10 μg mRNA. 24 h after injection, the mice were injected intraperitoneally with VivoGlo™ Luciferin in PBS at 150 mg kg^−1^ and imaged within 20 min using IVIS Lumina XR III.

### 2.12 *In vivo* delivery of PBAE-DOX/IL-12 mRNA to the mouse melanoma model

Female C57BL/6J mice were inoculated with 3.5 × 10^5^ B16F10 cells on the back skin. When tumours reached ∼50–200 mm^3^, they were intratumorally injected with PBAE-DOX/IL-12 at 10 μg mRNA and either 25 or 4 μg of doxorubicin 3 times, on days 1, 4 and 8. Mice were sacrificed on day 9, and tumours were flash frozen for analysis. ∼50 mg of the tumour was measured and homogenised in 500 μL of T-PER buffer containing a protease inhibitor cocktail. IFNγ and IL-12 were quantified using ELISA and normalised using the BCA assay. Doxorubicin was extracted and quantified from tumours as described by Bellary *et al.*:^31^ ∼100 mg of tumour was homogenised in 300 μL of cell lysis buffer (0.25 M sucrose, 5 mM Tris-HCl, 1 mM MgSO_4_, 1 mM CaCl_2_, pH 7.6). A standard curve was generated by spiking tumour from a PBS-treated mouse with hydrophobic doxorubicin prior to homogenization. To extract doxorubicin, 200 μL of tumour lysates including serial dilutions of spiked control was added to 100 μL of 10% Triton X-100, 200 μL of water and 1 mL of acidified isopropanol (0.75 N HCl) and incubated overnight at −20 °C. The next day, the samples were vortexed and centrifuged at 2000*g* for 15 min and fluorescence was measured on a microplate reader at 480 nm emission and 560 nm excitation.

### 2.13 Statistical analysis

All statistical analyses were performed using GraphPad Prism 10 except for Fig. 4E, where LAS X was used.

## 3. Results and discussion

### 3.1 PBAE formulated in phosphate buffered saline condenses doxorubicin and mRNA into positively charged nanoparticles

PBAEs are typically formulated in acidic buffer to increase amine protonation and electrostatic interaction with polyanions such as mRNA.^18–21^ However, this also increases aqueous solubility through increased hydrogen bonding of protonated amines, reducing the apparent hydrophobicity of the PBAE. Doxorubicin base readily complexes with hydrophobic polymers due to the strong hydrophobic interaction.^32^ To maximise hydrophobic interaction with doxorubicin, we investigated whether phosphate buffered saline (PBS, pH 7.4) would allow for spontaneous self-assembly of PBAE particles. When dissolved in typical sodium acetate buffer (NaOAc, 25 mM, pH 5.2), PBAE at 1 mg mL^−1^ was a clear solution (Fig. S1); however, when dissolved in PBS, the PBAE solution became turbid indicating reduced aqueous solubility (Fig. S1). When analysed by DLS, particles with a relatively small and uniform diameter of ∼155 nm and a polydispersity index (PDI) of 0.14 were observed (Fig. S1). To investigate whether the driving force for particle formation was increased hydrophobicity, we dissolved PBAE in PBS containing 50% acetonitrile (ACN), which can reduce hydrophobic interactions and increase the solubility of hydrophobic polymers. We found that no particles formed, confirming that hydrophobic interaction is necessary for the self-assembly of PBAE (Fig. S2). In contrast, when PBAE was dissolved in PBS containing 10% urea to reduce the formation of hydrogen bonding, particles were observed, and size remained unchanged compared to PBAE in PBS only. A constant correlation coefficient curve was observed, suggesting that hydrogen bonding does not contribute to PBAE aggregation. Furthermore, when PBAE was dissolved in a concentrated 10× PBS solution, the correlation coefficient curve shifted to the right, indicating that larger particles formed at increased ionic strength and salt concentration (Fig. S2). Together, these data suggest that although the self-assembly of PBAE is driven by hydrophobic interactions between polymer chains, polymer ionisation is important to prevent the formation of large aggregates through electrostatic repulsion.

**Fig. 1:**
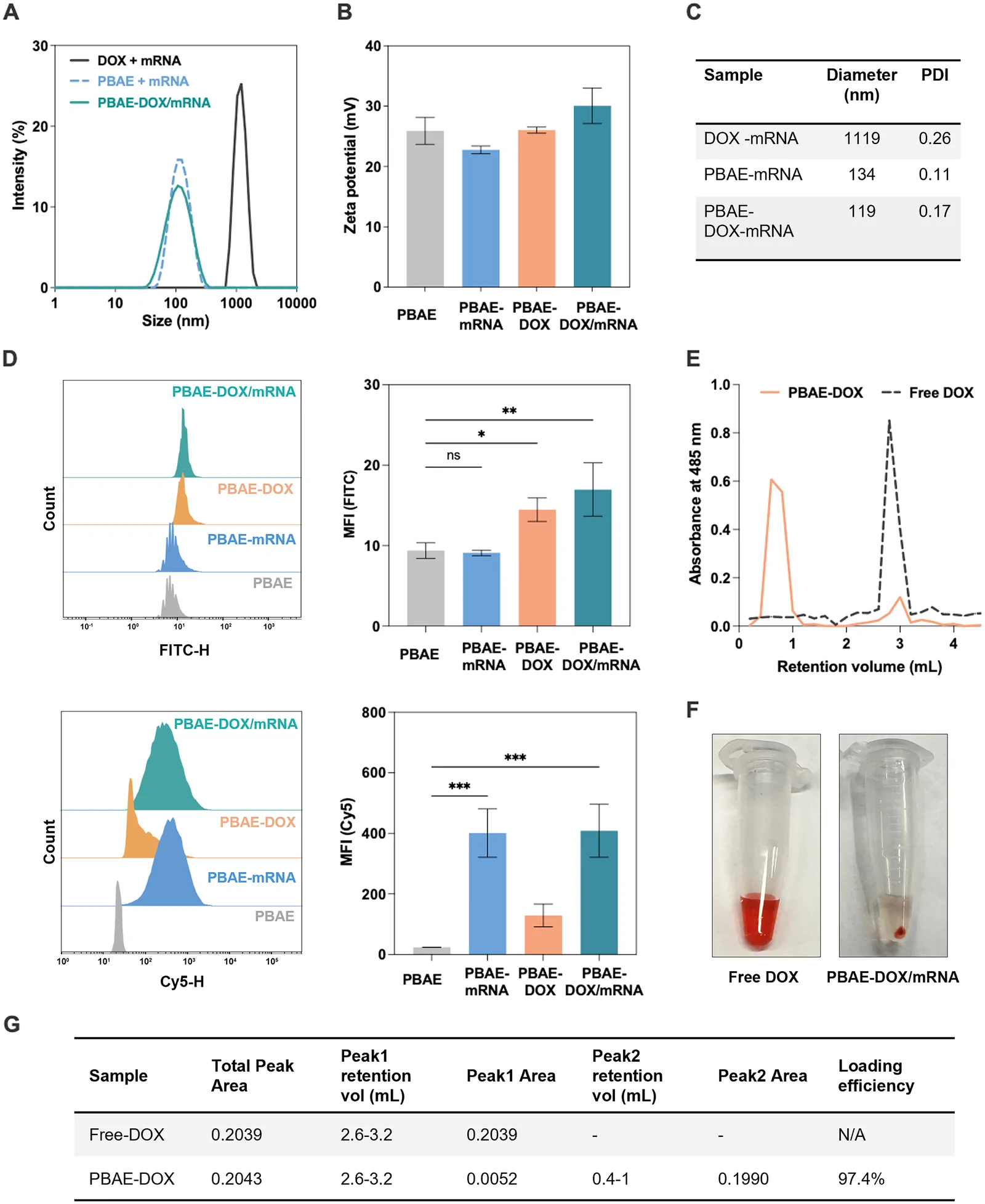
PBAE condenses doxorubicin and mRNA into positively charged nanoparticles when formulated in PBS. (A) Hydrodynamic size distribution, (B) zeta potential and (C) *Z*-Ave diameter and polydispersity index as measured by dynamic light scattering. (D) Characterisation of mRNA and DOX loading in PBAE-DOX/mRNA nanoparticles by NanoFCM. Data are presented as counts normalised to mode ± SD for *n* = 3 individually prepared batches of particles and analysed using one-way ANOVA, followed by Tukey's multiple comparison test. *p < 0.05, **p < 0.01, ***p < 0.001. (E) Size exclusion chromatography showing retention volumes of free DOX *vs.* PBAE nanoformulated-DOX. (F) Appearance of DOX (red) after high-speed centrifugation when free in solution or complexed with PBAE and mRNA. (G) Quantification of total peak areas observed in SEC encapsulation data.

Based on these results, PBS was selected as the solvent for DOX complexation with PBAE, yielding particles with a size of approximately 116 nm (Fig. S3A and B). However, in cell culture, the PBAE-DOX nanoparticles aggregated, most likely due to the absence of an anionic counterion to stabilise the electrostatic complexation (Fig. S3C). Subsequently, we attempted to complex PBAE with mRNA and DOX. To prevent spontaneous aggregation before mixing with mRNA, DOX and PBAE were first dissolved in DMSO and then added to mRNA diluted in PBS so that complexes would only form upon exposure to mRNA. Unlike free DOX and mRNA, which yielded large particles of approximately 1 μm (Fig. 1A), when the two cargos were complexed with PBAE, particle diameter reduced to approximately 120 nm (Fig. 1A). The zeta potential of PBAE-mRNA complexes increased from 26.04 mV without DOX, to 30.05 mV with DOX, potentially due to the additional positive charge from the amino group of DOX (p*K*_a_ = 9.93)^33^ (Fig. 1B). Furthermore, PBAE-DOX/mRNA nanoparticles showed a reduced polydispersity index (0.17) compared to DOX-mRNA (0.26), which supports the formation of monodisperse particles (Fig. 1C).

Although DLS confirmed particle size, the technique cannot confirm that DOX and mRNA are both associated with the PBAE polyplex. Therefore, nano-flow cytometry (NanoFCM) was employed to quantify DOX and Cy-5 tagged mRNA encapsulation by PBAE at the single-particle level (Fig. 1D). DOX autofluorescence was measured *via* the FITC channel and mRNA *via* the Cy5 channel. DOX loading was confirmed by a significantly higher mean fluorescence intensity (MFI) in the FITC channel when complexed with PBAE, compared to PBAE-only or PBAE-mRNA. Similarly, PBAE complexed with mRNA with or without DOX showed significantly enhanced Cy5 fluorescence intensity compared to PBAE or PBAE-DOX, indicating successful co-loading of DOX and mRNA with PBAE. Notably, there was no significant difference in Cy5 MFI between PBAE-mRNA and PBAE-DOX/mRNA particles, indicating that the presence of DOX did not affect mRNA loading (Fig. 1D), which is consistent with the zeta potential results.

To measure the loading efficiency of DOX with PBAE, SEC was employed to quantify the amount of free DOX eluted.^34^ Free DOX was first eluted through the column to determine its retention volume, which was around 3 mL (Fig. 1E). When PBAE-DOX was eluted, two peaks were observed: one at 3 mL, indicating the level of free DOX, and another larger peak at around 0.75 mL of PBAE complexed with DOX. This difference could also visually be observed after high-speed centrifugation of either free or PBAE complexed DOX, with only the latter forming a pellet (Fig. 1F). The Area Under the Curve (AUC) of the peaks was calculated and the loading efficiency of DOX in PBAE was found to exceed 97% (Fig. 1G). The gel retardation assay confirmed that mRNA was effectively complexed with the PBAE-DOX/mRNA nanoparticles, as indicated by the absence of the mRNA band during electrophoresis (Fig. S4). These results demonstrate that hydrophobic, positively charged PBAE can effectively complex the hydrophobic small molecule doxorubicin and mRNA to form monodisperse nanoparticles.

### 3.2 Sub-cellular distribution of Cy5-FLuc mRNA and doxorubicin in B16F10 melanoma cells

**Fig. 2:**
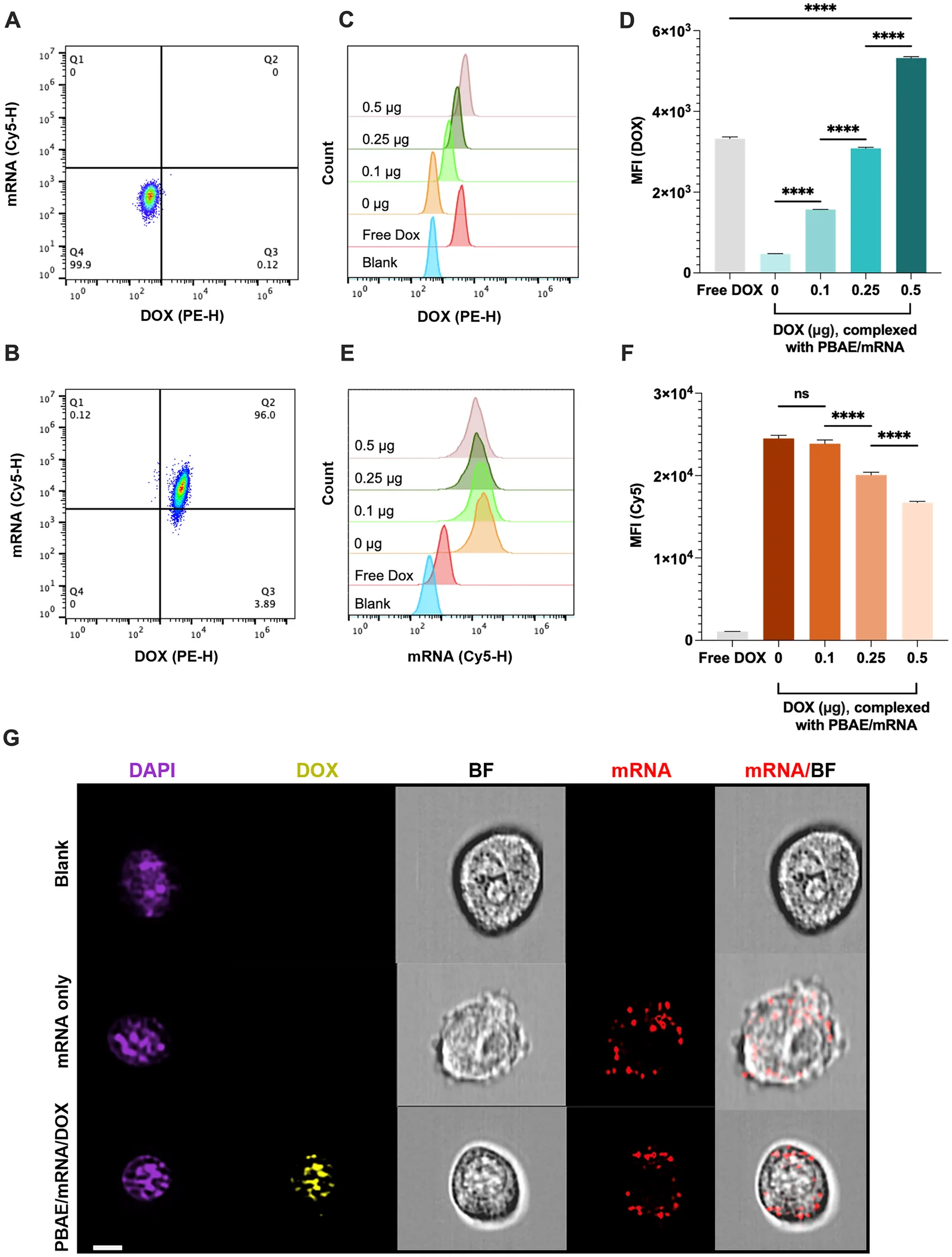
Imaging flow cytometry showing sub-cellular localisation of Cy5-FLuc mRNA in the cytoplasm and doxorubicin in nuclei of B16F10 cells. (A and B) Gating strategy for flow cytometry showing representative panels for (A) untreated ‘blank’ cells and (B) cells treated with PBAE, 0.5 μg DOX and 100 ng mRNA. (C and D) Histogram and mean fluorescence intensity (MFI) of DOX and (E and F) Cy5-mRNA in B16F10 cells when PBAE is complexed with 100 ng mRNA and DOX at varying doses. (G) Imaging flow cytometry showing uptake and localisation of mRNA in the cytoplasm and DOX in the nucleus of B16F10 cells; 100 ng of mRNA and 0.5 μg of DOX with PBAE were delivered per well, scale = 7 μm. Data are presented as ± SD for *n* = 3 individually prepared batches of particles, each with 3 technical repeats. Analysis using one-way ANOVA followed by Tukey's multiple comparison test, ****p < 0.0001.

After confirming the effective loading of both DOX and mRNA by PBAE, we sought to investigate the cellular uptake of DOX and mRNA. Flow cytometry (FCM) showed that after transfecting B16F10 cells with PBAE-DOX/Cy5-mRNA complexes, over 95% of cells were double-positive for both DOX and mRNA at 4 hours (Fig. 2B and Fig. S5A), demonstrating that PBAE enables the simultaneous delivery of DOX and mRNA (Fig. 2A–D). When the dose of DOX increased from 0.1 μg per well to 0.5 μg per well, the intensity of DOX increased significantly, confirming dose-dependent cellular uptake. When compared to 0.5 μg of free DOX, the equivalent DOX dose complexed with PBAE resulted in greater DOX intensity, indicating that the nanoformulation improved cellular uptake (Fig. 2E). Conversely, mRNA cellular intensity decreased with increasing DOX dose (Fig. 2F). Determining subcellular localization is important for mRNA, which functions in the cytoplasm, while DOX functions in the nucleus. Imaging flow cytometry was used to visualize whether Cy5-tagged mRNA and DOX can reach their site of action after uptake (Fig. 2G and Fig. S5B). Imaging FCM confirmed effective uptake of both DOX and mRNA by the cells, consistent with previous FCM results. Notably, the mRNA was localized in the cytoplasm, while DOX was mainly distributed in the nucleus. These findings further support the co-delivery of both mRNA and DOX by PBAE.

### 3.3 Delivery of PBAE-DOX/mRNA exhibits DOX dose-dependent cytotoxicity in melanoma cells

**Fig. 3:**
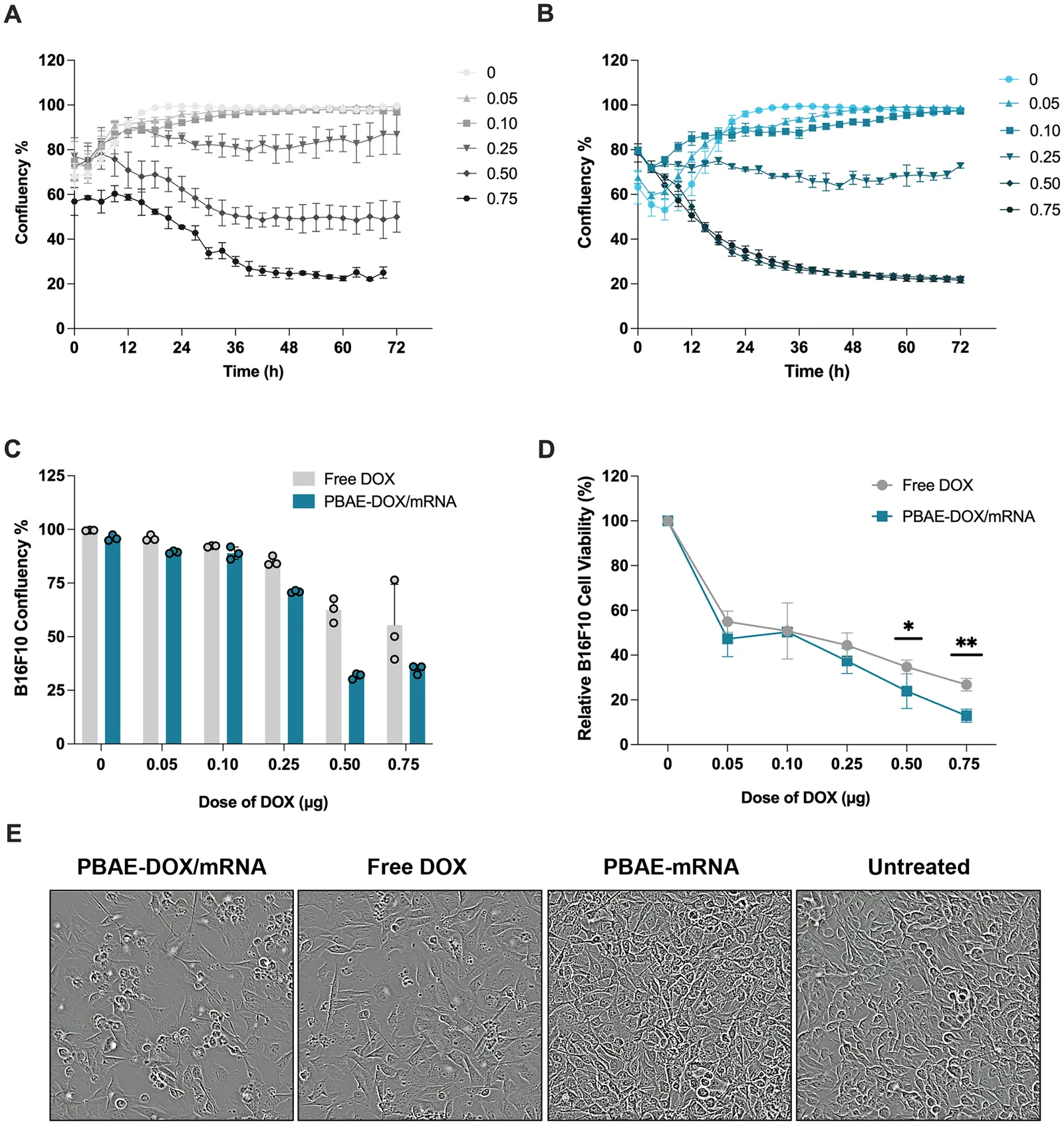
PBAE-DOX/mRNA exhibits DOX dose-dependent toxicity in the melanoma cancer cell line. B16F10 cells were treated with free DOX or PBAE-DOX/FLuc-mRNA at varying DOX doses and mRNA for 4 hours. Cell confluency (%) over 72 hours of (A) free DOX-treated and (B) PBAE-DOX/mRNA-treated cells. (C) Cell confluency (%) at 24 hours. Data are shown as means ± SD of *n* = 3 technical repeats (A–C). (D) Relative cell viability at 24 hours as measured by the Presto Blue assay, *n* = 3 biologically independent replicates. Data are normalised to an untreated control and analysed by one-way ANOVA, **P < 0.01, *P < 0.05. (E) Morphology of B16F10 cells 24 h following treatment with PBAE complexed with both mRNA and DOX or PBAE-mRNA only or free DOX at 0.25 μg. PBAE and mRNA doses were kept constant at 5 μg and 0.1 μg.

After confirming that PBAE enabled intracellular delivery of both mRNA and DOX to melanoma cells, we proceeded to explore the dose-dependent cytotoxicity of DOX when complexed with PBAE and luciferase mRNA compared to free DOX. B16F10 cells were treated with a dose range of 0.05–0.75 μg of free DOX or PBAE complexed DOX/mRNA, and cell confluency was tracked using live-cell imaging over 72 hours, and viability was assessed by metabolic activity. Both free DOX and PBAE-DOX/mRNA exhibited a dose-dependent reduction in cell confluency (Fig. 3A and B and Fig. S6). PBAE alone and PBAE-mRNA only (without DOX) did not reduce cell confluency compared to the non-treated control (Fig. S6). Interestingly, at 24 h, PBAE-DOX/mRNA exhibited higher cytotoxicity as observed by live-cell imaging and the cell viability assay as compared to equivalent doses of free doxorubicin (Fig. 3C–E). This difference was more pronounced at higher concentrations, where 0.75 μg of DOX loaded in PBAE/mRNA nanoparticles reduced B16F10 cell viability to 12.9% (SD = 2.7) compared to 26.6% (SD = 3.3) when free DOX was delivered at the same dose (Fig. 3D). A similar trend was observed in the EMT6 breast cancer cell line (Fig. S7).

### 3.4 Co-delivery of FLuc mRNA and DOX leads to simultaneous protein production and cytotoxicity

To establish whether reporter mRNA and doxorubicin co-delivery can achieve simultaneous protein production and cytotoxicity, B16F10 and EMT6 cells were transfected with Firefly Luciferase mRNA and increasing doses of doxorubicin. Translation to reporter protein was quantified by bioluminescence at 24 hours, and cell viability relative to non-transfected control was determined by metabolic activity.

**Fig. 4:**
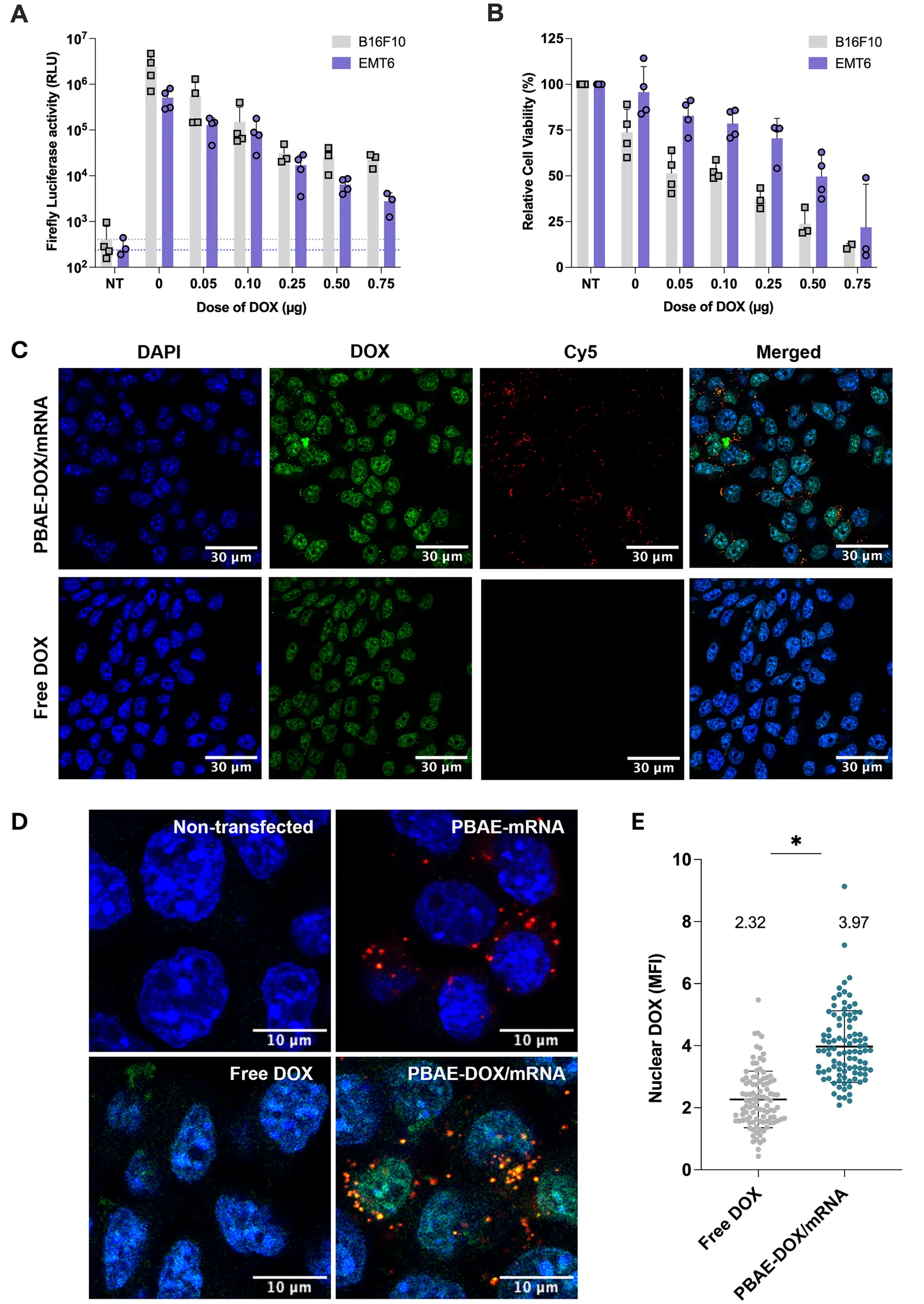
PBAE-DOX/FLuc mRNA reporter translation in breast (EMT6) and melanoma (B16F10) cell lines and B16F10 cell co-localisation. Cells were treated with free DOX or PBAE-DOX/Cy5-tagged Fluc-mRNA at 0.05–0.75 μg DOX for 4 hours. (A) Translation of luciferase mRNA at 24 hours using the luciferin substrate assay. (B) Relative viability at 24 h compared to a non-treated control was determined by the PrestoBlue assay. Data are shown as means ± SD of *n* = 3–4 independent biological replicates. (C and D) Uptake of PBAE complexed with 0.4 μg of DOX (green) and 0.2 μg of Cy5-Fluc mRNA (red), or PBAE-Cy5-mRNA or free DOX at 6 hours in B16F10 cells following a 4-hour treatment. Nuclei are stained with DAPI (blue). Co-localised regions of mRNA and DOX appear yellow in the merged image. (E) Mean fluorescence intensity of doxorubicin within nuclei at 6 h. Data are shown as means ± SD of 18–40 nuclei from *n* = 3 images for each group. Statistical analyses were performed using an unpaired two-tailed *t*-test. *=*p* < 0.05.

As expected, increasing the dose of doxorubicin decreased mRNA-encoded protein production, likely due to increasing levels of cell death (Fig. 4A and B) and potentially due to reduced intracellular mRNA levels at higher DOX doses, observed during flow cytometry (Fig. 2F). However, even at the highest doxorubicin concentrations, where up to an 88% decrease in B16F10 cell viability was observed, effective mRNA translation two orders of magnitude above the control could be detected (2.4 × 10^4^ RLU) compared to non-transfected cells (4.16 × 10^2^ RLU) (Fig. 4A).

Next, sub-cellular localization of Cy5-FLuc mRNA and DOX was evaluated by confocal microscopy, which showed accumulation of the molecules at their respective sites of action in B16F10 cells (Fig. 4C). The cells were fixed at 6 h post-transfection, and nuclei were stained with DAPI. At 6 h, doxorubicin primarily localised within nuclei, whereas the Cy5 signal appeared in the cytoplasm, confirming observations from imaging FCM (Fig. 2G). Importantly, punctate regions of higher DOX intensity appear co-localized, with the Cy5 signal in the *peri*-nuclear and cytosolic regions, which cannot be seen in the free DOX control (Fig. 4D), building on evidence that complexation of DOX and mRNA occurs within the same nanoparticles. Interestingly, the mean fluorescence intensity of doxorubicin within nuclei was on average 1.7-fold higher in the B16F10 cells treated with PBAE-DOX/mRNA compared to the cells treated with free DOX (Fig. 4E). This supports the potential for nanoformulated DOX to reach the nucleus to a greater extent than free DOX.

### 3.5 Co-delivery of doxorubicin and *IL-12* mRNA leads to simultaneous cytotoxicity and translation of biologically active IL-12

**Fig. 5:**
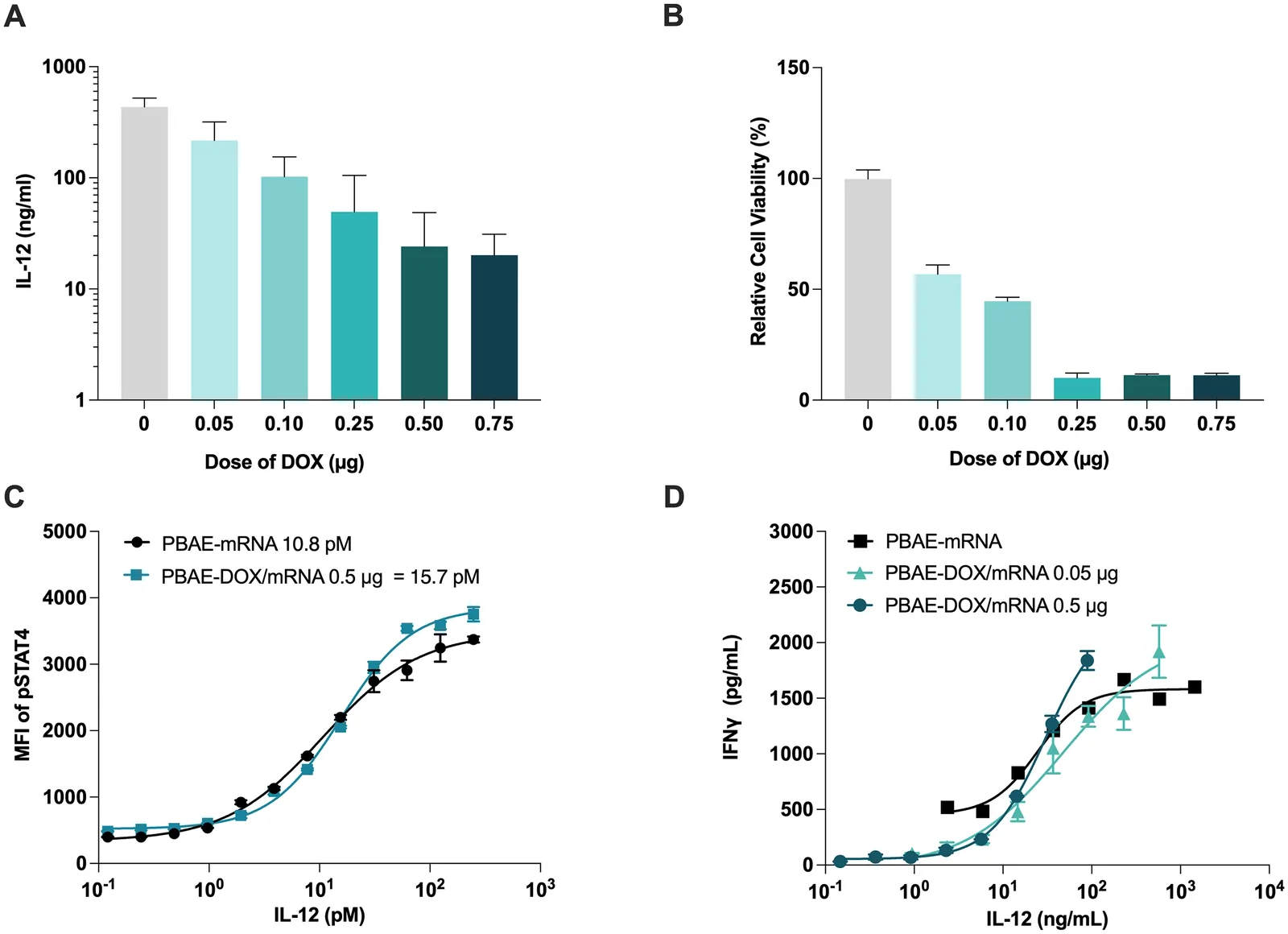
Delivery of PBAE-DOX/*IL-12* mRNA to B16F10 cells produces secreted IL-12 protein that is biologically active in primary mouse splenocytes. B16F10 cells were treated with PBAE complexed with 100 ng of *IL-12* mRNA and 0.05–0.75 μg of DOX for 4 hours. (A) IL-12 protein was determined at 24 h post-transfection by ELISA. Data are shown as means ± SD from 2 independent biological replicates with *n* = 3 technical replicates each. (B) Cell viability at 24 h relative to no DOX (0) control. (C and D) Primary mouse splenocytes were activated for 3 days with CD28/CD3/IL-2 and stimulated with the supernatant from B16F10 cells transfected with PBAE complexed with 100 ng *IL-12* mRNA and DOX at two doses, or PBAE-*IL-12* mRNA without DOX. (C) STAT4 phosphorylation of CD3+ splenocytes following 15-minute stimulation with the cell supernatant and (D) IFNγ secretion following 24 h stimulation. Data are shown as means ± SD of *n* = 3 technical replicates.

After confirming that PBAE could facilitate intracellular delivery of DOX and mRNA leading to simultaneous cell death and reporter translation, we extended validation of the platform using therapeutically relevant *IL-12* mRNA and assessed cytokine function. B16F10 cells were transfected with PBAE co-formulated with DOX and *IL-12* mRNA, and secreted IL-12 protein was quantified at 24 hours. Cell viability and cytotoxicity were measured by metabolic activity and lactate dehydrogenase (LDH) release assays, respectively. Consistent with previous results with reporter mRNA, there was efficient translation of *IL-12* mRNA with a DOX dose-dependent reduction in translation efficiency and cell viability. At 24 hours post-transfection, co-delivery of 100 ng *IL-12* mRNA with 0.05 μg DOX yielded 216.8 ng mL^−1^ (±82.5) of IL-12 protein (Fig. 5A). The relative cell viability of B16F10 cells decreased to 56.8% (±3.7) as determined by the Presto Blue assay (Fig. 5B) and LDH release (Fig. S10).

To assess the biological function of secreted IL-12 protein translated from mRNA, we stimulated CD28/CD3-activated primary mouse splenocytes^35^ with serial dilutions of IL-12 in supernatant secreted from transfected B16F10. STAT4 phosphorylation was measured after 15 minutes of stimulation *via* flow cytometry and IFNγ secretion at 24 h by ELISA. The translated IL-12 induced dose-dependent phosphorylation of STAT4 (Fig. 5C) and downstream secretion of IFNγ (Fig. 5D) from mouse splenocytes. The presence of DOX in the nanoparticle did not significantly affect the functionality of the secreted IL-12 protein, as determined by EC_50_ (Fig. 5C).

### 3.6 PBAE facilitates *in vivo* co-delivery of *IL-12* mRNA and doxorubicin to the melanoma mouse model

**Fig. 6:**
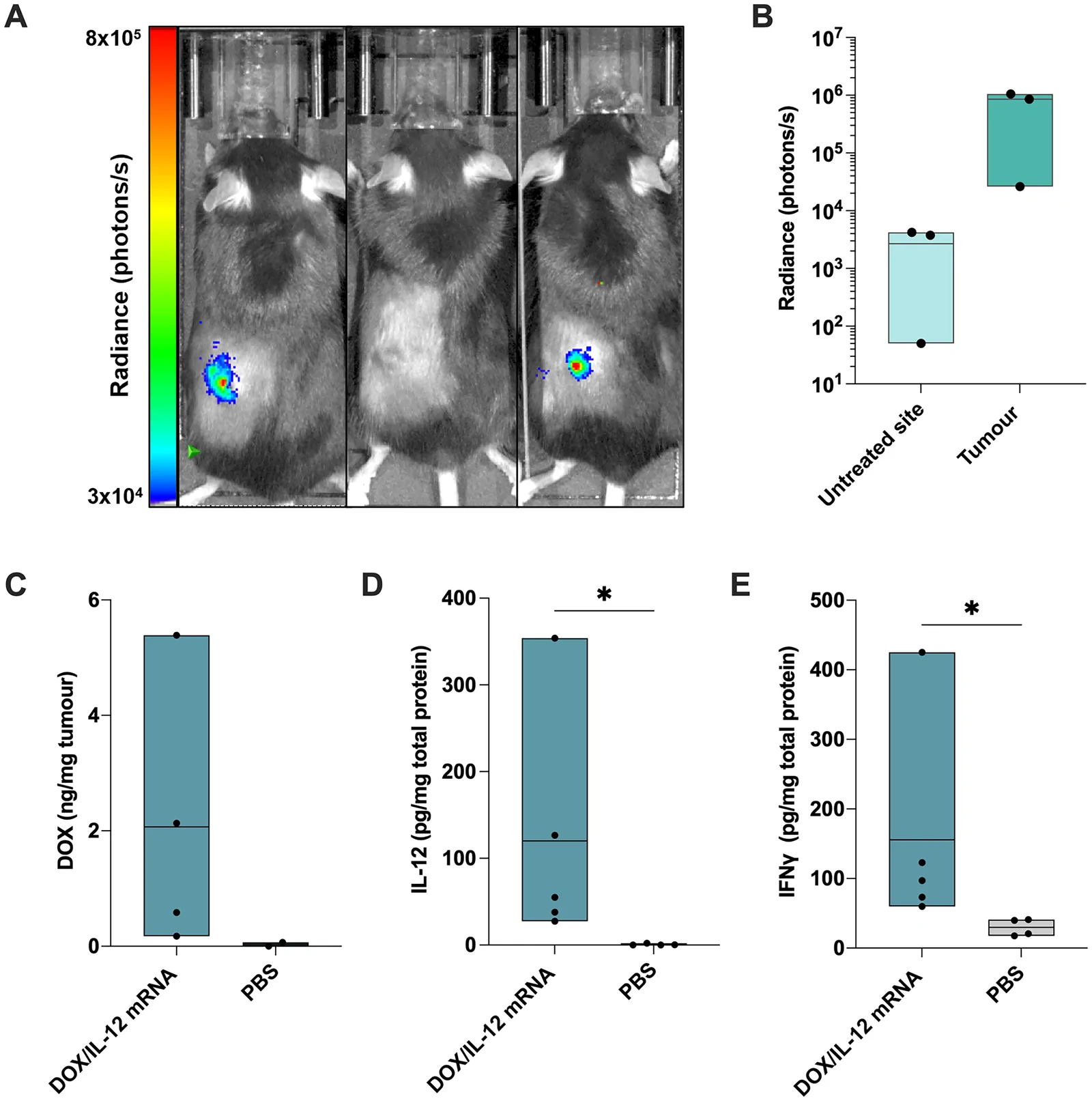
Pilot study to establish co-delivery and tolerability of PBAE-DOX/*IL-12* mRNA after intratumoural administration to mouse melanoma. (A and B) C57BL/6 mice were inoculated with B16F10 cells subcutaneously. Once tumours reached between 50 and 200 mm^3^, they were injected intratumourally with 10 µg of FLuc mRNA complexed with PBAE. Luminescence was imaged 24 hours post-injection. Data are presented as floating bars (min–max) with the median for *n* = 3. (C–E) C57BL/6 mice with B16F10 subcutaneous tumours of ∼50–200 mm^3^ were injected intratumourally with 10 μg *IL-12* mRNA complexed with DOX and PBAE, or saline control (PBS), 3 times over 9 days as outlined in Fig. S12. Tumour levels of (C) DOX, (D) IL-12 and (E) IFNγ quantified 24 h after the final injection. Data are presented as floating bars (min–max) with the median; *n* = 4–5 for DOX/*IL-12* mRNA and *n* = 2–4 for PBS harvested at humane endpoints. IL-12 and IFNγ concentrations were normalised to total protein. Statistical significance among the two groups was analysed by the Mann–Whitney *U* test, *=*p* < 0.05.

PBAE complexed mRNA has previously been delivered intratumorally to a B16F10 murine model^36^ but to further characterise biodistribution, we injected PBAE-encapsulated FLuc mRNA or a buffer control into B16F10 tumours *in vivo* and visualized luminescence 24 h post-injection. A strong signal localised to the tumour was observed in 2 out of 3 treated mice (Fig. 6A and B). Previous studies have demonstrated variable translation of mRNA following intratumoral injection with other delivery systems.^37,38^ In the B16F10 model in particular, this deviation may be due to variability in the necrotic core, which can impact exogenous gene expression.^39^

Finally, we sought to understand the feasibility and tolerability of repeated co-delivery *in vivo* following intratumoural injection. Mice bearing B16F10 tumours were injected with PBAE-DOX/*IL-12* mRNA three times over a nine-day period (Fig. S11) and the levels of DOX, IL-12 and IFNγ were measured from tumour lysate 24 hours after the final injection. Compared with the PBS control, tumours treated with PBAE-DOX/*IL-12* mRNA showed increased levels of DOX, IL-12 and IFNγ (Fig. 6C–E). No signs of weight loss or acute toxicity were observed in treated animals following the injections (Fig. S12). This suggests that both DOX and *IL-12* mRNA were successfully delivered to tumour cells, resulting in downstream IL-12 translation and biological activity and an improved probability of survival (ns, *p* > 0.05) (Fig. S12), supporting further dose escalation and therapeutic efficacy studies.

## 4. Discussion

There is growing interest in mRNA-based immunotherapies for their transient and localised expression, and the synergistic effects of DOX and IL-12 are well-established.^21,23^ The combination of DOX and *IL-12* mRNA in a single nanocarrier presents an unexplored opportunity. This study establishes a polymeric nanoparticle platform with the capacity to co-formulate the two cargos for effective delivery to murine melanoma.

Concurrent association of the payloads within individual particles was confirmed by nanoFCM, and confocal imaging indicated co-localisation of DOX and mRNA in punctate regions of a melanoma cell line. Although the proportion of loading for each cargo was not determined, there was a reproducible functional response for DOX and mRNA when co-formulated with PBAE. In addition, SEC revealed that PBAE achieved high levels of DOX loading comparable to clinically relevant formulations.^40^

A notable aspect of this work is that nanoparticle assembly was achieved at physiological pH in phosphate-buffered saline, where PBAE self-assembly appears to be driven largely by hydrophobic interactions while retaining sufficient ionization to facilitate incorporation of hydrophobic DOX alongside anionic mRNA. Importantly, compared to the free DOX control, the nanoformulated DOX resulted in greater intensity of the chemotherapeutic in the nucleus, which correlated with greater cytotoxicity at a lower dose, a finding that supports previous reports of harnessing nanomedicine for improved intracellular accumulation.^41^ In contrast, the level of mRNA translation reduced with increasing DOX dose, which is expected, given the reduced cell viability. This observation identifies a window in which protein production, which relies on cellular activity, can occur alongside chemotherapy-induced cell death.

Intratumoral injection of PBAE-encapsulated DOX/*IL-12* mRNA in a syngeneic mouse model of melanoma led to tumour retention of DOX and localised IL-12 protein. Mice tolerated repeated dosing, which is of significance, as secreted IL-12 could potentially enter the circulation, which is associated with systemic toxicity.^42^ However, intratumoral administration is not universally applicable with many tumors being inaccessible, and even within injectable lesions, elevated interstitial pressure, and variable necrosis^39^ can hinder uniform exposure.^43,44^ Our luciferase biodistribution study also indicated variability of mRNA translation, suggesting that broader utility of this approach should consider alternative administration routes.

In summary, this study successfully demonstrated the feasibility of an advanced chemo-immunomodulatory strategy of cytokine mRNA with a cytotoxic payload and opens avenues for next-generation combination cancer therapies.

## Conflicts of interest

JI is a shareholder of Kan Therapeutics. AP and HS are inventors on patents related to biomaterials for gene delivery.

## Ethical statement

All animals were handled in accordance with the Animals Scientific Procedures Act 1986 and under a project license approved by the United Kingdom Government Home Office. The project was overseen by the ethics committees at Imperial College London.

## Supplementary Material

PM-003-D6PM00136J-s001

## Data Availability

The data supporting this article have been included as part of the supplementary information (SI). Supplementary information: turbidity of PBAE in buffer, correlation coefficient and dynamic light scattering of DOX and PBAE, gel electrophoresis of PBAE complexed with DOX, imaging flow cytometry, cell confluency data, cytotoxicity, confocal imaging, flow cytometry gating strategy, and weight loss and survival data. See DOI: https://doi.org/10.1039/d6pm00136j.
