# Supplementary material for: Co-formulation of IL-12 mRNA and doxorubicin in polymeric nanoparticles for simultaneous delivery in murine melanoma
Source: RSC Pharm. 2026 Jul 1. Online ahead of print. doi: 10.1039/d6pm00136j (PMC13370319; doi:10.1039/d6pm00136j)
Supplement: PM-OLF-D6PM00136J-s001 [file PM-OLF-D6PM00136J-s001.pdf]

Supplementary Information (SI) for RSC Pharmaceutics.
This journal is © The Royal Society of Chemistry 2026

Co-formulation of IL-12 mRNA and doxorubicin in polymeric nanoparticles for simultaneous delivery in murine melanoma.

Elina Tanskanen[1,2], Hongning Sun[2,1], Kai-Chun Cheng[1], Jun Ishihara[2], Asha K. Patel[1]

[1]National Heart and Lung Institute, Imperial College London, London, UK.

[2]Department of Bioengineering, Imperial College London, London, UK

Supplementary Information

Supplementary methods

<u>Turbidity test (S1)</u>

PBAE stock solution was diluted by PBS or NaOAc buffer (25 mM, pH 5.2) to 1 mg mL^{-1}, and turbidity determined by absorbance at 600 nm.

<u>Dynamic Light Scattering (S2-4)</u>

PBAE was dissolved in DMSO at 50 mg mL^{-1}. 1 µL of PBAE stock solution was diluted by 150 µL of different buffers (including PBS, PBS with 10% urea, 10 x PBS, 25 mM pH 5.2 NaOAc buffer, and PBS with 50% ACN) and transferred into a microcuvette for particle size measurement by Malvern Zetasizer Pro.

<u>Encapsulation of mRNA within PBAE-DOX/mRNA nanoparticles (S5)</u>

Gel retardation assay was used to confirm encapsulation of mRNA within PBAE-DOX/mRNA nanoparticle. Nanoparticles containing various concentrations of doxorubicin and 500 ng of mRNA encapsulated by PBAE were stained with 6x gel loading dye and run on a 1% agarose gel with SYBR Safe stain at 100V for 1h. Retardation of mRNA was visualized against Riboruler High Range Ladder.

Supplementary figures

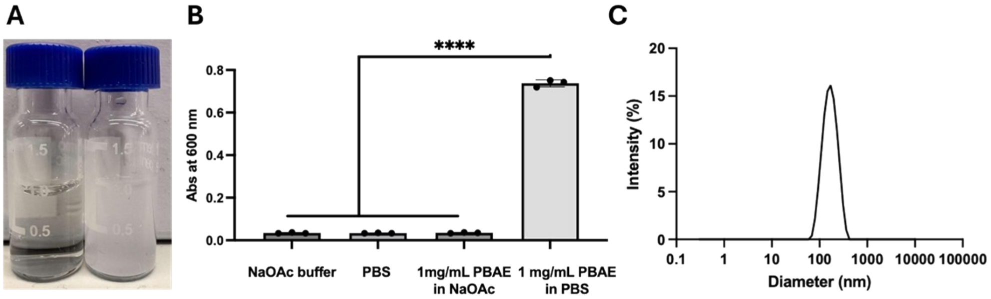

Supplementary Figure 1. (A) Image showing colour difference between PBAE dissolved in NaOAc buffer (left) and PBS (right). **(B)** Absorbance measurements of PBAE dissolved in NaOAc or PBS buffer measured at 600 nm. Data is presented as ± SD, for n=3 independent nanoparticle preparations, analysed by one-way ANOVA with Tukey's multiple comparison test. **(C)** Size distribution of PBAE in PBS as measured by dynamic light scattering.

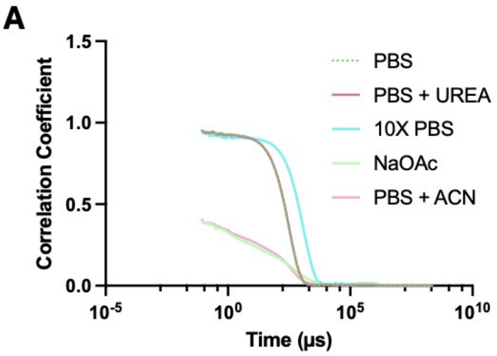

B

	Size	SD (Size)	PDI	SD (PDI)
PBS	205.8	9.036	0.0793	0.02256
PBS+ Urea	214.3	17.5	0.06074	0.008179
10x	1008	149.3	0.09877	0.05594

Supplementary figure 2. (A) Correlation Coefficient and **(B)** Size and PDI of PBAE in different solutions as measured by dynamic light scattering. Data shown for n=1 measurement.

A

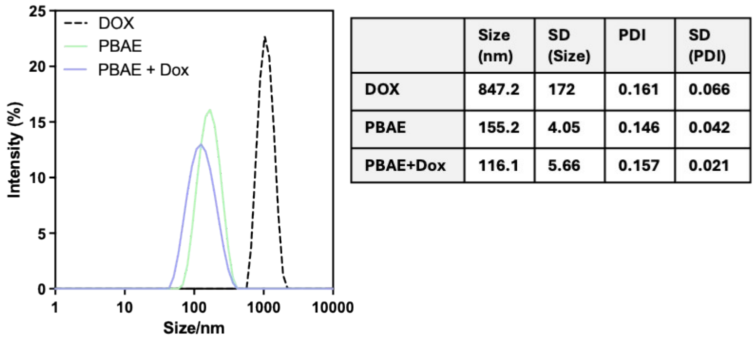

B

	Size (nm)	SD (Size)	PDI	SD (PDI)
**DOX**	**847.2**	**172**	**0.161**	**0.066**
**PBAE**	**155.2**	**4.05**	**0.146**	**0.042**
**PBAE+Dox**	**116.1**	**5.66**	**0.157**	**0.021**

C

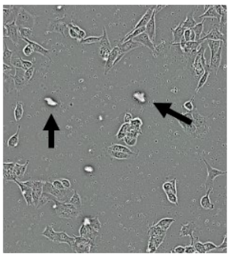

Supplementary figure 3. (A) Size distribution and **(B)** Polydispersity index (PDI) of DOX, PBAE and PBAE-DOX in PBS as measured by dynamic light scattering. Data is shown as mean ±SD for n=3 measurements. **(C)** Aggregation of PBAE-DOX particles on Cellcyte X ~2h post-treatment

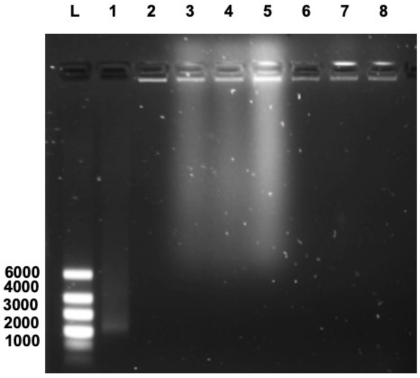

Lane	Sample
L	Ladder
1	Naked mRNA
2	PBAE- mRNA 50:1 w/w
3	PBAE-DOX.HCl 50:5 w/w
4	PBAE-DOX.HCl/mRNA 50:10:1 w/w
5	PBAE-DOX.HCl/mRNA 50:5:1 w/w
6	PBAE-DOX 50:5 w/w
7	PBAE-DOX/mRNA 50:10:1 w/w
8	PBAE-DOX/mRNA 50:5:1 w/w

Supplementary figure 4. Gel retardation assay showing encapsulation of mRNA within PBAE-mRNA and PBAE-DOX/mRNA nanoparticles. DOX= DOX base, DOX.HCL= DOX salt.

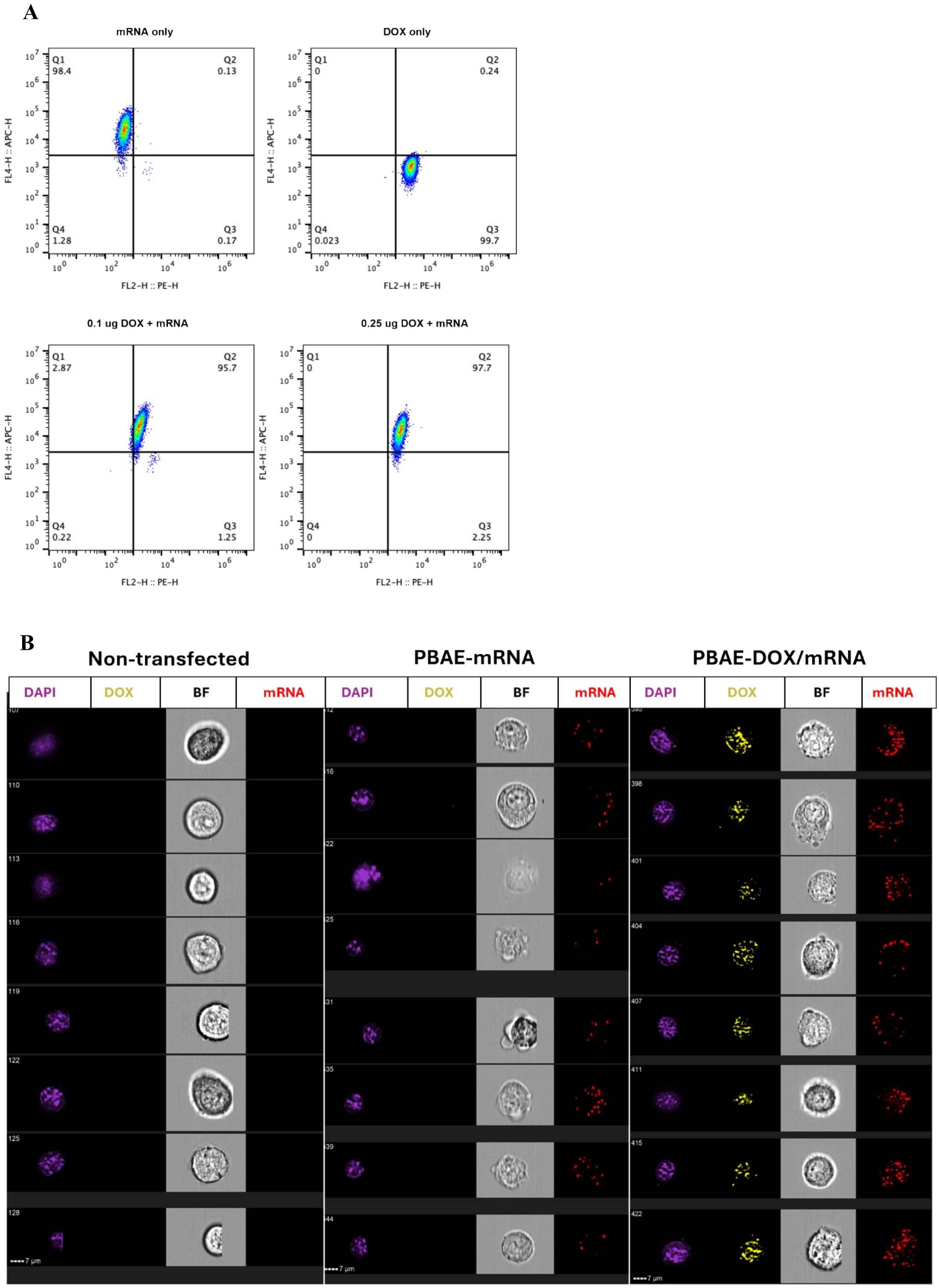

Supplementary figure 5. (A) Flow cytometry of B16F10 cells 4 hours post-treatment with free DOX only, or PBAE+DOX, and PBAE+mRNA**. (B)** Imaging flow cytometry showing uptake and localisation of cy5 tagged mRNA (red) in cytoplasm and DOX (yellow) in nucleus (blue) of B16F10 cells. 100 ng of mRNA and 0.5 µg of DOX were delivered per well. Scale = 7 µm.

A

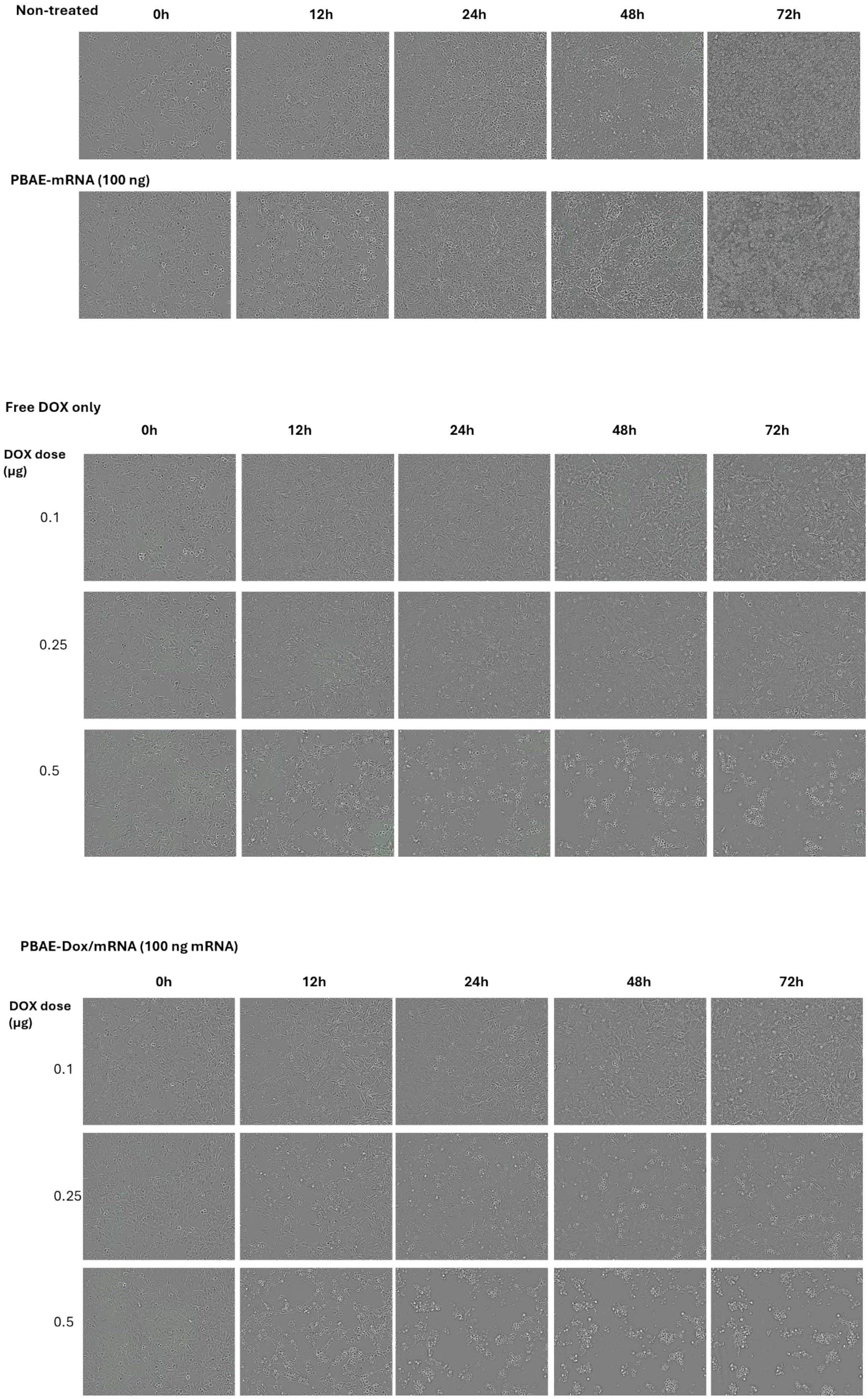
Non-treated
0h
12h
24h
48h
72h
PBAE-mRNA (100 ng)
Free DOX only
0h
12h
24h
48h
72h
DOX dose (μg)
0.1
0.25
0.5
PBAE-Dox/mRNA (100 ng mRNA)
0h
12h
24h
48h
72h
DOX dose (μg)
0.1
0.25
0.5

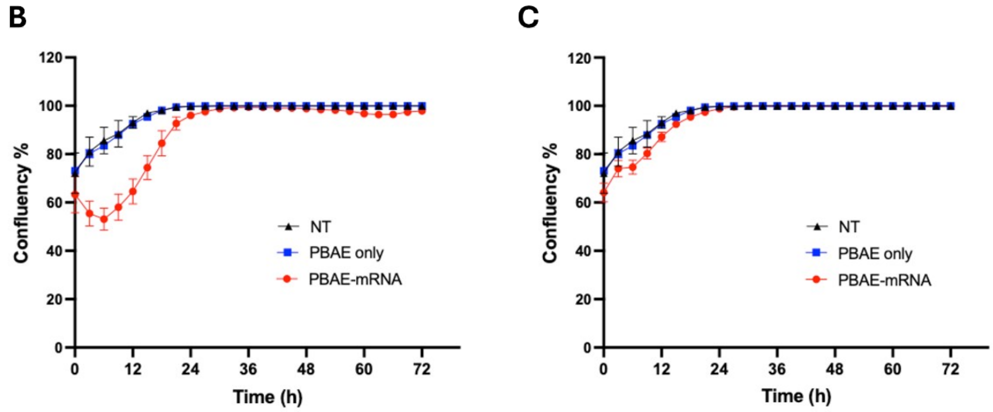

Supplementary figure 6. (A) Images of B16F10 cell confluency from CellCyte after treatment with varying doses of DOX with 100 ng mRNA and 5 µg PBAE, or controls. Cell confluency (%) of **(B)** B16F10 and **(C)** EMT6 cells following treatment with 5 µg PBAE alone or complexed with 100 ng of FLuc mRNA. Data is shown as mean ±SD for n=3 technical replicates.

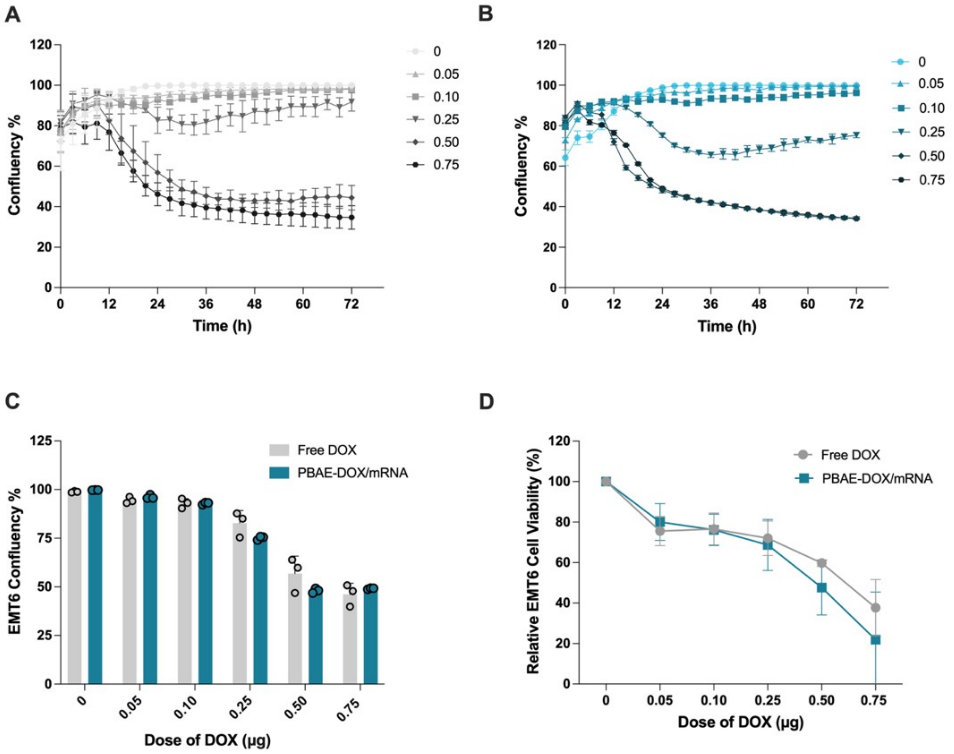

Supplementary Figure 7. PBAE-DOX/mRNA exhibits dose-dependent toxicity on EMT6 breast cancer cell line. Cells were treated with free DOX or PBAE-DOX/FLuc-mRNA at 0.05 – 0.75 µg DOX for 4 hours. Cell confluency (%) of **(A)** Free DOX -treated and **(B)** PBAE-DOX/mRNA -treated cells as observed by Cellcyte X over 72 hours. **(C)** Cell confluency (%) quantified by Cellcyte X at 24 hours. **(D)** Relative cell viability at 24 hours as measured by Presto Blue assay. Data are shown as means ±SD of n=3 technical repeats (A-C) or n=3 biologically independent replicates (D). PBAE and mRNA doses were kept constant for at 5 µg and 0.1 µg, respectively (50:1 PBAE: mRNA w/w). For (D) data is normalised to untreated control.

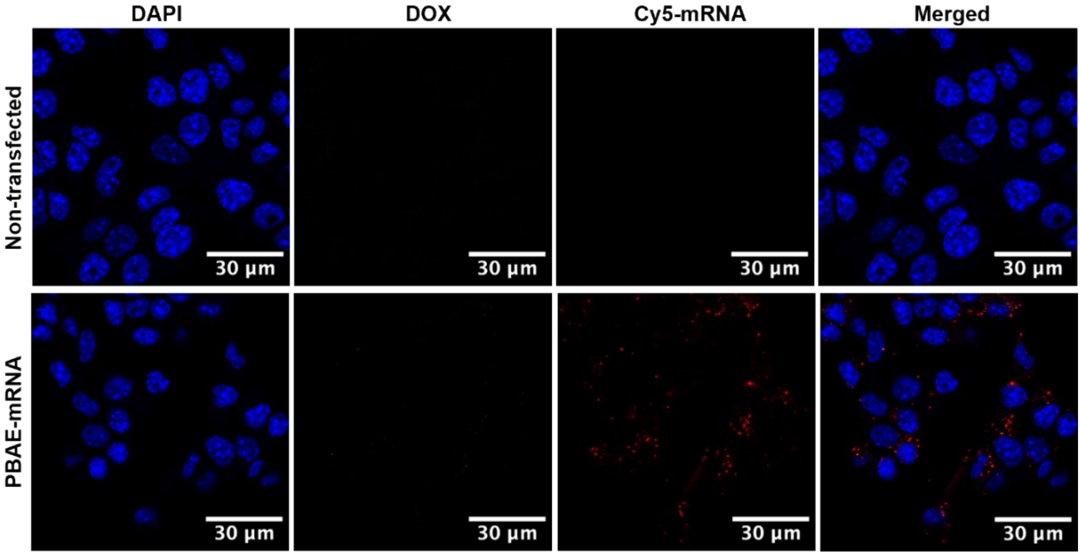

Supplementary figure 8. Confocal images showing uptake of PBAE-encapsulated cy5-FLuc mRNA (0.2 μg) compared to non-transfected control at 6 hours in B16F10 cells following 4-hour treatment. Nuclei are stained by DAPI (blue).

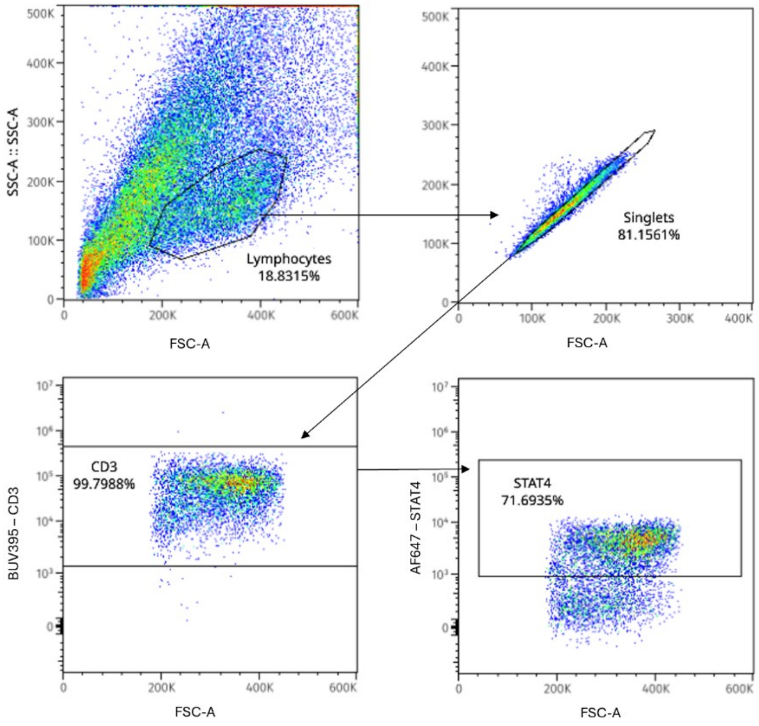

Supplementary figure 9. Gating strategy for pSTAT4 assay in Figure 5.

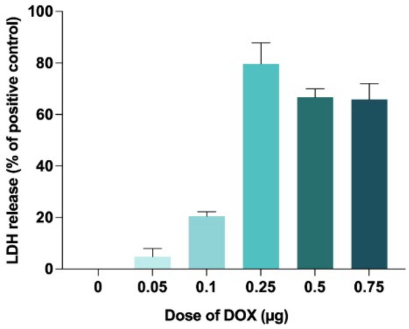

Supplementary figure 10. LDH release from B16F10 cells at 24h after treatment with 5 µg of PBAE complexed with 100 ng of *IL-12* mRNA and 0.05-0.75 µg of DOX for 4 hours.

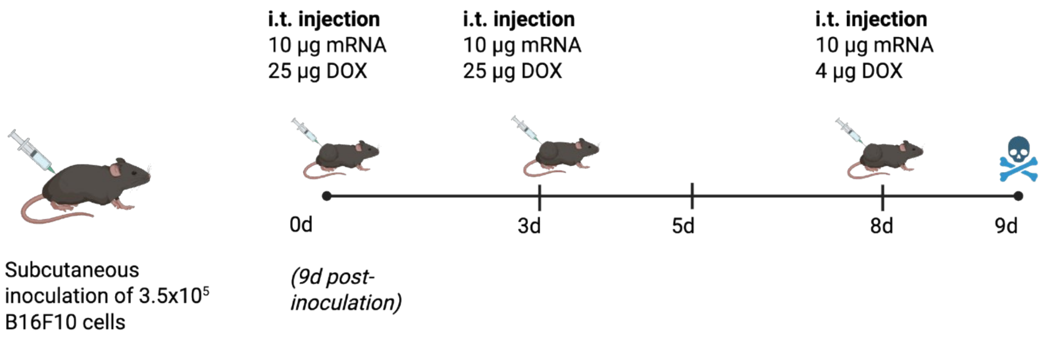

Supplementary figure 11. Treatment regimen for Figure 6.C-E B16F10 study. Created with Biorender.com. Available from: https://www.biorender.com/20en4jy

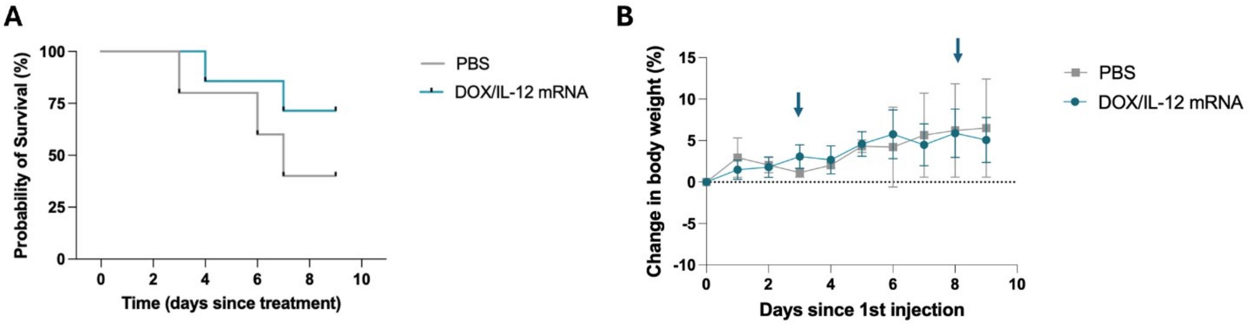

Supplementary figure 12. Changes in body weight (%) following intratumoral injection of PBAE-DOX/*IL-12* mRNA or PBS. Timing of 2nd and 3rd injections are indicated with arrows. Mice that reached humane endpoint prior to the end of the study are excluded. Data shown as means ±SEM for n=5 (PBAE-DOX/*IL-12* mRNA) or n=2 (PBS).
